# Gleanings from the German

**Published:** 1875-07-01

**Authors:** H. Gradle


### GLEANINGS FROM THE GERMAN.

By H. Gradle, M.D.

Dr. Sale has introduced remedies into the cavity of the uterus by enclosing them in gelatine capsules and bringing them into place by means of forceps (*Allg. Wien. Med. Zeit.*, No. 19).

Guillaumet (*Journal de Ther.*, 1874, No. 3) recommends bisulphide of carbon very highly in atonic ulcers, the surface of which he paints with the same, afterward dusting on subnitrate of bismuth or starch powder, and covering it with dry charpie, according to the amount of secretion. By the rapid evaporation cold is produced, and the granulations become pale at first but soon redden, while an intense pain, lasting twenty to sixty seconds, occurs. This is sometimes followed by local anæsthesia for hours. The violent pain, however, is lost in repeated applications. Improvement occurs very rapidly, even in the most obstinate cases.—*Centralblatt fuer Chir.*, No. 20.

According to Miremond (*Journ. de Med. et de Chir.*), five to ten grammes of red precipitate ointment applied with friction, for one to two minutes, around and to the surface of a carbuncle, causes immediate amelioration and resolution within two to three days, without suppuration.

G. Marcacci (*Lo Sperimentale*, 1875, Fasc. 1) performed laryngotomy, on account of the existing dyspnœa, in the case of a little girl whose trachea was partly occluded by a bean which had entered. The blood, however, running down the trachea, increased the dyspnœa, and all attempts to seize the bean were unsuccessful. At this moment, Marcacci, Jr. introduced a feather into the trachea, in the hope of causing a reflex expiratory effort, which really did occur, and extruded the bean. Still the child made no further respiratory movements, and only by the maintenance of artificial respiration for five or six minutes was life preserved.

A somewhat anæmic though otherwise healthy woman had suffered from an abortion two years ago, since which time a violent uterine catarrh existed, with extensive ulceration around the os. Tr. iodine, perchloride of iron, nitrate of silver, and concentrate sulphuric acid had been used in vain, until A. di' Bernardo (*Gaz. Hebdom.*, 1874) employed a solution of hydrate of chloral—two parts in twenty-five. After painting the ulcer with this five to six times, it was healed in the course of a fortnight. In two similar cases, the success was the same. B. recommends to continue the application for a few days after the ulcers have healed.

After various attempts by different physicians to render the urine acid by internal administration of different acids, which all, however, passed into the urine in the shape of neutral salts,

Gossehn and Robin recollected the peculiarity of benzoic acid, which leaves the system as hippuric acid. The same is the case with cinnamonic and salicylic acids, and the acids derived from Balsam Tolu and B. Peru. A daily dose of thirty to ninety grains of this agent has no unpleasant consequences except a slight momentary burning and subsequent dryness of the pharynx. As the drug is soluble only in 607 parts of cold water, it is best administered in a mucilage of sugar and water. In five to nineteen days, on the average about the seventh or eighth day, the quantity of phosphate of calcium, pus and blood, as well as the fetor of the ammoniacal urine of cystitis, begin to diminish gradually. The authors have arrived at the following conclusions:

1. Before undertaking a serious operation on the urinary organs, it is advantageous to obviate, or at least diminish, the alkalinity of the urine.

2. This purpose is best attained by the internal use of flores benzols, and the balsams containing salicylic or cinnamonic acids.

3. The hippuric acid contained in the urine acts in the following manner:

*a.* Hippurate of ammonia is less poisonous than the corresponding carbonate.

*b.* Hippuric acid retards the fermentation of the urine, and hence also the formation of carbonate of ammonia.

*c.* It prevents likewise lithrasis and consecutive cystisis.

4. Hence the drug is indicated in purulent cystitis.—*Allg. Med. Cent. Zeit.*, No. 40.

